# Sirtuins as Important Factors in Pathological States and the Role of Their Molecular Activity Modulators

**DOI:** 10.3390/ijms22020630

**Published:** 2021-01-10

**Authors:** Ewa Maria Kratz, Katarzyna Sołkiewicz, Adriana Kubis-Kubiak, Agnieszka Piwowar

**Affiliations:** 1 Department of Laboratory Diagnostics, Division of Laboratory Diagnostics, Faculty of Pharmacy, Wroclaw Medical University, 50-556 Wroclaw, Poland; katarzyna.solkiewicz@umed.wroc.pl; 2 Department of Toxicology, Faculty of Pharmacy, Wroclaw Medical University, 50-556 Wroclaw, Poland; adriana.kubis-kubiak@umed.wroc.pl (A.K.-K.); agnieszka.piwowar@umed.wroc.pl (A.P.)

**Keywords:** sirtuins expression, sirtuin activators, sirtuin inhibitors, sirtuins in pathological conditions, oxidative stress

## Abstract

Sirtuins (SIRTs), enzymes from the family of NAD^+^-dependent histone deacetylases, play an important role in the functioning of the body at the cellular level and participate in many biochemical processes. The multi-directionality of SIRTs encourages scientists to undertake research aimed at understanding the mechanisms of their action and the influence that SIRTs have on the organism. At the same time, new substances are constantly being sought that can modulate the action of SIRTs. Extensive research on the expression of SIRTs in various pathological conditions suggests that regulation of their activity may have positive results in supporting the treatment of certain metabolic, neurodegenerative or cancer diseases or this connected with oxidative stress. Due to such a wide spectrum of activity, SIRTs may also be a prognostic markers of selected pathological conditions and prove helpful in assessing their progression, especially by modulating their activity. The article presents and discusses the activating or inhibiting impact of individual SIRTs modulators. The review also gathered selected currently available information on the expression of SIRTs in individual disease cases as well as the biological role that SIRTs play in the human organism, also in connection with oxidative stress condition, taking into account the progress of knowledge about SIRTs over the years, with particular reference to the latest research results.

## 1. Introduction

**Figure 1:**
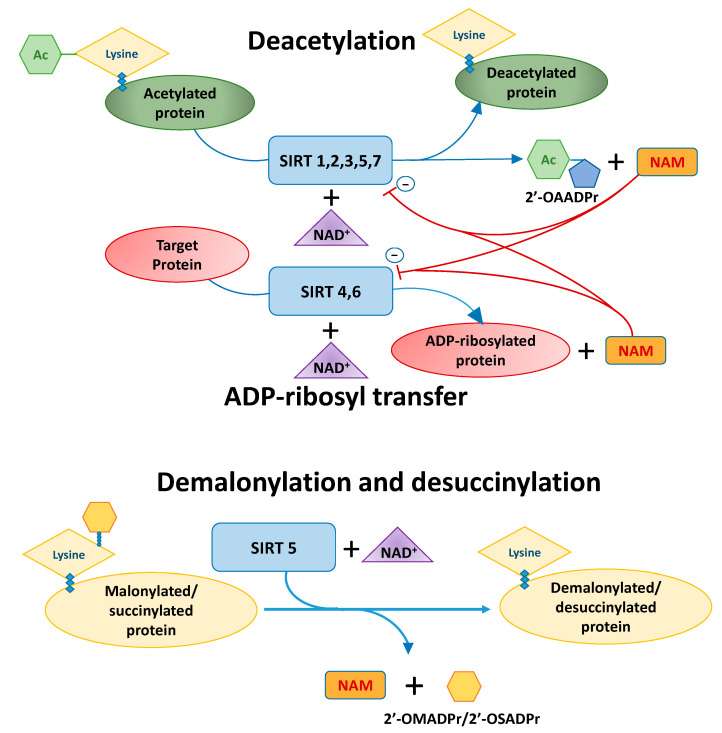
Enzymatic activity of the SIRTs. NAD^+^ is a substrate for all SIRTs in reaction of nicotinamide (NAM) production. In the deacylation reaction, NAD^+^ receives the acetyl (Ac) group from the protein. The adenine dinucleotide moiety from NAD^+^ is linked to various residues on the target protein in ADP-ribosyl transfer. SIRT1, 2, 3, 5 and 7 show the lysine deacetylation activity of target proteins in which the NAD^+^ coenzyme is used to produce NAM and 2′-O-acetyl-ADP-ribose (2′-OAADPr). SIRT4 exhibits only ADP-ribosyl transferase activity, and NAD^+^ is used as a donor of the ADP-ribose group for target proteins. ADP-ribosyl transferase activity shares with SIRT6. SIRT5 uses NAD^+^ as a cofactor in the demalonylation and desuccinylation of target proteins, generating NAM and 2’-O-malonyl-ADP-ribose (2′-OMADPr) and 2′-O-succinyl-ADP-ribose (2′-OSADPr), respectively. Modification based on Morigi et al. [4,5].

The change in gene expression accompanying gene inheritance does not have to be related to the direct modification of the genetic code, but may also take place through an extra-genomic mechanism, which is related to the ability to modify the histone proteins included in the chromatin, strongly bound to the DNA helix. The most important mechanisms of these changes are the processes of acetylation and deacetylation of lysine, an abundant component of histones. Post-translational modifications of histone proteins may also occur as a result of their methylation, phosphorylation or ubiquitination, but the acetylation/deacetylation mechanism is the most common [1]. The two main families of enzymes involved in the processes of histone acetylation and deacetylation (histone acetyltransferases, HATs and histone deacetylases, HDACs, which include sirtuins, SIRTs) [2,3], also play an important role in the process of post-translational modifications [3]. The main biochemical processes that take place in the human body with the participation of SIRTs are shown in Figure 1.

SIRTs are enzyme proteins that belong to the nicotinamide adenine dinucleotide (NAD^+^) family of histone deacetylases. The structure, biological functions, target location, and the main substrates of catalytic activity have been discussed in detail in numerous reviews [6,7,8,9]. Table 1 shows the structure of the SIRTs and their target location.

SIRTs are present in all living organisms, and in addition to regulating the degree of histone acetylation, they are also involved in many other processes that take place within the cell, including involvement in the regulation of the cell cycle and energy metabolism, as well as in the process of cell differentiation, growth and apoptosis. The role of SIRTs has also been proven in the response of cells to different types of stress, e.g., oxidative, energetic or induced by ultraviolet radiation or through action on various target substrates [3,15]. Many scientific studies focus on the analysis of the activity changes of SIRTs in pathological conditions, ranging from neurodegenerative diseases, through liver ailments, digestive system maladies, infertility and insulin resistance, to neoplastic states and oxidative stress conditions [16,17,18,19]. Due to such a wide range of activities, SIRTs have aroused particular interest among researchers in recent years. Latest observations give high hopes for the use of these proteins for diagnostic purposes as markers of disease progression, but also for therapeutic purposes based on their activation or inhibition, in order to increase the effectiveness of patients’ treatment.

This study collects information from latest reports while also taking into account the advancement of knowledge about SIRTs over the years. It characterizes the most important currently recognized SIRTs modulators that may have an activating or inhibitory impact. The participation of SIRTs and the degree of their expression in selected disease entities is also presented and discussed in the context of their main roles in the organism.

### 1.1. Sirtuins and Oxidative Stress

Growing evidence provides information that supports the substantial role of SIRTs family in the regulation of cellular homeostasis, in particular metabolism and oxidative stress [20,21]. An environment of oxidative stress is said to be a consequence as well as a reason for conditions such as metabolic syndrome or obesity. It seems that, as SIRTs controls metabolic responses, it can also have an impact on the general disruption of redox cellular homeostasis [22,23,24]. The fact that SIRTs deacetylase activity is dependent on NAD^+^, a key redox signaling molecule, may support the idea that sirtuins may be important players in regulating cellular antioxidant and redox signaling (ARS) pathways. The coenzyme NAD^+^ mediates redox reactions by carrying electrons from one reaction to another, being an oxidizing agent, which accepts an electron, as well as may converts into its reduced form, NADH, and vice versa [25]. The overwhelmed generation of ROS can also affect SIRTs’ activity at genetic, posttranslational modification, and protein-protein interaction levels. For example, oxidative stress increases expression and, as a consequence, the activity of SIRT 1, leading to higher levels of their deacetylated substrates such as transcription factor p53, forkhead box proteins O or peroxisome proliferator-activated receptor gamma coactivator 1 alpha (PGC1α). As a result of the aforementioned alterations, stronger antioxidative response is detected via SOD2, glutathione peroxidase or catalase expression [26,27,28,29,30]. On the other hand, some in vitro data shows that exposition to high doses of H_2_O_2_ (100 µM or 250 µM) significantly decreased SIRT1 protein activity and gene expression [31,32,33]. Posttranslational modifications triggered by oxidative stress, such as phosphorylation of SIRT at different C-terminal residues or sumoylation, has been shown to upregulate its enzymatic activity [34]. Kinases such as Cdc2 or Casein Kinase II, regulated by oxidative stress, are known to phosphorylate SIRT on the C-terminus domain [35,36]. 5’AMP-activated protein kinase—a crucial regulator of the redox state of the cell, the biological activity of which is regulated by oxidative stress—can also phosphorylate SIRT, mainly by affecting binding to its protein inhibitor [37,38]. Mutations of different sites on SIRT1 affect cell cycle progression, trigger a conformational change reflected in enzymatic activity alterations, or lead to yet unexamined functional consequences [39,40,41]. Oxidative modifications of SIRTs are less well studied. Yang et al. [42] have shown that SIRT1 undergoes covalent oxidative modification by cigarette smoke-derived oxidants and aldehydes, leading to its inactivation and degradation. There is also a study that reports treatment of HEK293 cells with nitrosoglutathione resulting in nitrosylation of nuclear SIRT1 and inhibited deacetylation of PGC1α, a known regulator of the expression of mitochondrial antioxidants such as SOD2 [43]. Treatment of recombinant SIRT6 with the peroxynitrite donor SIN-1 revealed nitration of the enzyme and diminished activity [44]. Another mechanism by which oxidative stress regulates the activity of different SIRTs is by altering their binding to regulatory proteins. In the case of SIRT1, it promotes the phosphorylation of protein (DBC1) deleted in breast cancer 1, which increases its affinity for SIRT1, leading to SIRT inhibition [45]. It is still unknown whether the active regulator of SIRT1—another regulatory protein—plays an active role in the mediation of response to oxidative stress in cells [46,47]. As NAD^+^ availability is key in the regulation of all SIRTs, the inhibition of major NAD^+^-consuming enzymes such as poly(ADP-ribose) polymerase 1 (PARP-1), or the genetic deletion or pharmacological inhibition of the protein CD38 (the main NAD glycohydrolase), activates SIRT1 and protects against metabolic and age-related diseases [48,49,50,51]. However, SIRT1 is the most well-studied member of the mammalian sirtuin family, also in context of its connection with oxidative stress, it is known that all of the mammalian sirtuins may be associated with oxidative stress signaling and antioxidant defense in the cell [24,25], with particular emphasis on sirtuins located in mitochondria which are involved in maintaining redox homeostasis in the cell. One of them is SIRT3 which full-length form resides in the nucleus but trans-locates to the mitochondria in response to stress, e.g., DNA damage [52]. SIRT3 is known to mediate the flow of mitochondrial oxidative pathways and therefore regulate the production of ROS [53], and can affect cellular health directly by influencing the production of ROS through modulation of enzymes involved in the mitochondrial oxidative phosphorylation (OXPHOS) pathway [54]. Human mitochondrial DNA (mtDNA) encodes for 13 proteins, which are known to play a role in regulating respiration and OXPHOS in the mitochondria, and is significantly more susceptible to oxidative damage than nuclear DNA [55]. In the cell, ROS generated by OXPHOS, ionizing radiation, or chemicals are responsible for DNA damage, including changing purine and pyrimidine bases to 8-oxo-7,8-dihydroguanine (8-oxoG). Importantly, SIRT3 has been shown to target the enzyme that repairs this DNA damage, human 8-oxoguanine-DNA glycosylase 1 (OGG1). SIRT3 has been shown to promote OGG1 by binding to it to prevent degradation and control its activation when DNA glycosylase becomes active. Moreover, SIRT3 has been found to be crucial in the repair of mtDNA, protecting the integrity of mitochondria, and protecting the cell from apoptosis under conditions of oxidative stress by mediating the activity and replenishment of OGG1 [56]. Thus, SIRT3 has an important role in protecting the cell against genotoxic stress and oxidative damage, underlining its importance in ARS. SIRT4 is also a mitochondrial sirtuin which the main function is ribosylation of adenosine diphosphate (ADP) [57,58]. Unlike other sirtuins, SIRT4 was initially thought to not have NAD^+^-dependent deacetylase activity. Recently, however, SIRT4 was found to have the ability to deacetylate lysine, allowing it to control leucine metabolism and insulin secretion [57]. Furthermore, some authors suggest that all the sirtuins, except SIRT4, play a key role in the reduction of mitochondrial oxidative stress throughout the caloric restriction process [59,60]. This, however, does not minimize the role played by SIRT4 in oxidative stress regulation in the cell. SIRT4 has been shown to be involved in the regulation of ROS production in mitochondria, although it is unclear if it affects the activation of antioxidant enzymes localized to the mitochondrial matrix [59,60]. Mitochondrial SIRT5 functions is deacetylation, demalonylation, and desuccinylation multiple proteins [58,61]. SIRT5 play a role in cellular metabolism, detoxification, regulation of oxidative stress, energy production, and mediation of the apoptosis pathway [62]. However, there is no definitive on the roles of SIRT5 in these processes, and many conflicting views seem to exist in this regard, it appears that its association with ROS and oxidative stress signaling is strong. SIRT5 is popularly known for the regulation of mitochondrial fatty acid oxidation, the urea cycle, and cellular respiration [63,64]. SIRT5 deacetylates and activates carbamoyl phosphate synthetase (CPS1), which catalyzes the first step of the urea cycle for the detoxification of ammonia [63] which is known to induce ROS production and decrease antioxidant GSH content [65], suggesting the indirect involvement of SIRT5 in managing oxidative stress. SIRT5 has also been shown to deacetylate cytochrome C, which is an essential component of the electron transport chain [66]. In a Liang et al. study [67], cells that were transfected with SIRT5 were found to have decreased levels of ROS, suggesting that SIRT5 suppresses the progression of oxidative stress conditions in the cell. Overall, it appears that SIRT5 play an important role in a response of cells to oxidative stress. However, this area of research still needs further efforts aimed at elucidating the exact mechanism of SIRT5 functions [24]. Delicate modifications to the expression and activity of SIRTs are crucial to maintain cellular homeostasis. Although it’s clear that SIRTs are modulated by oxidative stress, the molecular mechanisms that underlay this relation are not well understood. Active SIRTs can defend cells from ROS-induced destruction via their product, O-acetyl-ADP-ribose, which hinders mitochondrial ROS assembly and raises NADPH levels from the pentose phosphate pathway [24,68].

### 1.2. The Sirtuin-Nrf2 Interaction

Nuclear factor E2-related factor 2 (Nrf2) is generally recognized as a transcription factor activated by oxidative stress that induces the production of antioxidant proteins and supports the regulation of redox homeostasis. Nrf2, which is naturally bound to Kelch-like ECH-associated protein 1 in the cytoplasm, where it undergoes proteolytic degradation and rapid turnover, is then phosphorylated, and translocated to the nucleus. It binds to the antioxidant response element target genes and increases the transcription of a variety of anti-oxidative and multiple cytoprotective enzymes, including NADPH-quinone oxidoreductase 1, glutathione S-transferases, and heme oxygenase-1 [69,70]. The function of the Nrf2-antioxidant pathway is controlled by multiple factors, including the acetylation-deacetylation of Nrf2. While SIRT1 and NRF2 are typically believed to function via separate pathways, recent evidence suggests SIRT1 involvement in regulating the expression and activation of Nrf2 [24]. Kawai et al. [71] demonstrated that SIRT1-mediated deacetylation of the Nrf2 protein terminated the transcription of antioxidant genes in vitro. However, other studies have demonstrated that SIRT1 overexpression significantly promoted the nuclear accumulation, DNA binding and transcriptional activity of Nrf2 and Nrf2-mediated gene expression [72,73]. Some studies have shown that the transcriptional activity of Nrf2 can be regulated by SIRTs, which upregulate Nrf2 downstream gene expression of genes encoding superoxide dismutase and glutation [72,74]. Conversely, downregulation of SIRT1 expression significantly reduced Nrf2 protein expression. Others found that overexpression can deacetylate Nrf2 and surge the stability as well as stimulate the transport of Nrf2 to the nucleus. Moreover, it promotes the transcriptional activity of Nrf2 and improves the resistance of cells to oxidative damage. In mouse type II alveolar epithelial cells exposed to paraquat (herbicide strongly toxic to humans), the SIRT1/Nrf2 signaling pathway played a protective role. SIRT1 overexpression in these cells, induced by transfecting with a SIRT1 overexpression vector, led to a substantial increase in Nrf2 protein expression and activity, which in consequence upregulated the expression of Nrf2 downstream genes such as superoxide dismutase, glutation, catalase and heme oxygenase-1. The effect was inhibition of cell apoptosis. SIRT1 downregulation by transfecting it with SIRT1-targeting shRNA induced the production of malondialdehyde, which resulted in increased oxidative damage [75]. A group from P. Barnes laboratory [76] have analyzed the effects of sirtuin inhibition using sirtinol (a non-specific sirtuin inhibitor) on Nrf2 protein levels as well as its activity after stimulation with H_2_O_2_. Results show that increases in Nrf2 stability are not controlled by sirtuins but by class I and II histone deacetylases. A new study reported that SIRT2 deacetylates lysines 506 and 508 residues of Nrf2, resulting in a decrease in both total cellular and nuclear levels of Nrf2 (through its degradation). The diminution in nuclear Nrf2 is associated with a reduction in its transcription of the target gene Fpn1 [77]. Conversely, it has been shown on PC12 neuroblastoma cells that SIRT2 may positively regulate nuclear Nrf2 expression by modulating Akt phosphorylation. Additionally it was prevented by both SIRT2 siRNA and SIRT2 inhibitor—AGK2. SIRT2 siRNA also blocked the NADH-induced increases in glutamate cysteine ligase (GCL) and glutathione [78]. Another study on PC12 neuroblastoma cells showed that silencing can virtually abolish the effects of NAD^+^ on the Nrf2 activity. Moreover, the SIRT2 inhibitor AGK2 treatment attenuated the increases in the Nrf2 mRNA levels and the nuclear Nrf2 levels induced by NAD^+^ [24,79]. McDougald et al. [80] have interrogated the impact of SIRT1 or Nrf2 overexpression in experimental optic neuritis via adeno-associated virus gene transfer to retinal ganglion cells. Authors have examined whether the SIRT1 or Nrf2 gene augment the suppression of retinal ganglion cell death, optic nerve inflammation, and demyelination in a mouse model of multiple sclerosis. Transfection with the SIRT1 gene results in a positive trend in retinal ganglion cell survival. The Nrf2 gene transfer provided the most robust protective response with respect to total and regional retinal ganglion cell survival. There is also a study showing that SIRT1 deacetylated and reduced the ubiquitination level of Nrf2 in Sprague Dawley rat primary glomerular mesangial cells challenged with advanced glycation-end products. It also promoted Keap1/Nrf2/ARE anti-oxidative pathway activation, which exerts crucial inhibitory effects on the development of diabetic nephropathy, leading to a reduction in fibronectin and TGF-β1 levels. The diminution of Nrf2 blocked those effects of SIRT1. Interestingly, Nrf2 also positively regulated SIRT1 at the levels of protein expression and deacetylase activity [81].

**Figure 2:**
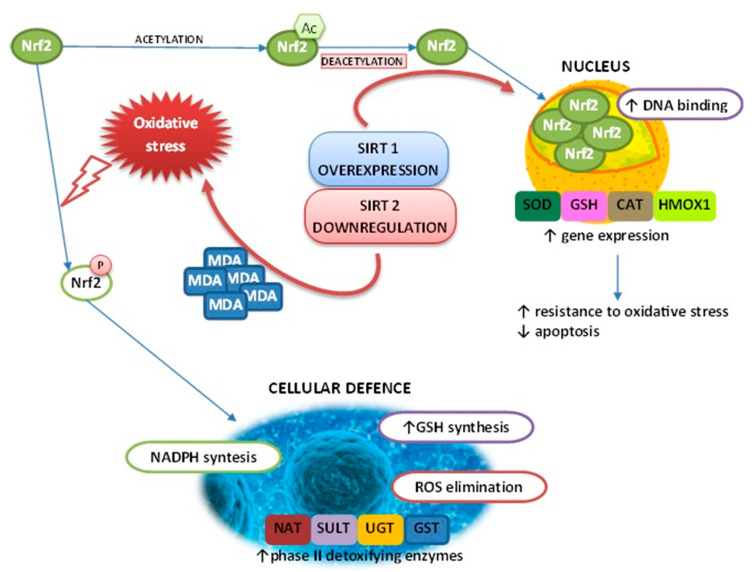
Schematic representation of the effect of SIRTs on Nrf2 metabolism. GSH—glutathione; GST—glutathione S-transferase; UGT—UDP-glucuronosyltransferase; NAT—N-acetyltransferase; SULT—sulfotransferase; SOD—superoxide dismutase; CAT—catalase; HMOX1—heme oxygenase-1; MDA—malondialdehyde.

It can be seen that the SIRT/Nrf2 pathway can antagonize oxidative damage by enhancing the cells’ antioxidant capacity. Further research is needed to clarify whether the regulation of Nrf2 may also have significant effects on defense mechanisms against oxidative stress, as Nrf2 is activated by oxidative stress and plays a crucial role in the transcriptional activation of the defense machinery. The above mentioned effect of SIRT on Nrf2 action is presented on Figure 2.

### 1.3. The Sirtuin-NF-κB Interaction

**Figure 3:**
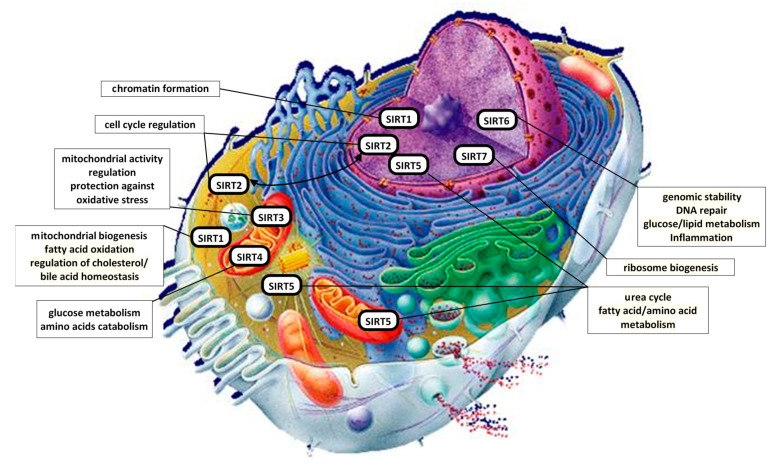
Cellular localization and main cellular mechanisms in which SIRTs participate [82].

Despite the somewhat misleading ‘histone deacetylase’ term, SIRTs amend a broad range of non-histone proteins and transcription factors including those responsible for anti-inflammatory response such as nuclear factor kappa-light-chain-enhancer of activated B cells (NF-κB). The NF-κB pathway has been proposed to be an universal promoter of the innate immunity and inflammatory response, as well as its activity plays a significant role in boosting neurodegeneration processes, ischemia and other diseases [82]. Overexpression of SIRT1 deacetylase and the addition of resveratrol blocked NF-κB-dependent gene transactivation stimulated by amyloid β (Aβ) and had strong neuroprotective effects in microglia BV2 and primary neuronal culture isolated from Sprague-Dawley rat pups [83]. The modulation of senescence via NF-κB is possible due to its interactions with SIRTs. While, overactive NF-κB signaling may lead to premature aging it was shown that SIRT6 tempers this process via deacetylation of histone H3 lysine 9 at NF-κB target gene promoter [84]. Despite vast spectrum of cellular mechanism and intracellular localizations (shown in Figure 3), most SIRTs inhibits NF-κB activity.

For example, SIRT1 and SIRT6 deacetylates RelA subunit of NF-κB, repressing its target promoters inhibiting apoptosis and cellular senescence [84,85]. SIRT1 also interacts with NF-κB’s transcriptional corepressor- transducin-like enhancer of split-1 (TLE-1) and deacetylates lysine 310 on p65 protein leading to diminution of NF-κB activity [86,87] SIRT3—a transcriptional target of NF-κB, was shown to regulate inhibitory effect of metformin on NF-κB activity in differentiated L6 skeletal muscle cells with induced insulin resistance [88]. In cigarette smoke extract (CSE)-treated human pulmonary microvascular endothelial cells overexpression of SIRT4 blocks the degradation of inhibitor of NF-κB and in consequence downregulated CSE-induced NF-κB activation [89]. Moreover, SIRT4 mitigated nuclear translocation and transcriptional activity of NF-κB in human umbilical vein endothelial cells suppressing endothelial inflammation processes [90]. SIRTs thus concurrently influence the pro-inflammatory and possibly toxic activities of NF-κB and modulates the stress resistance signals of insulin/IGF-I signaling [82,91]. Furthermore, SIRT1 also interact with an important partner of p53 protein and NF-κB—p300 by inhibiting its acetylating activity [92]. Protein p300 is a transcriptional co-activator which role is to arrest the interaction of histones with DNA through their acetylation. However, the impact of SIRT2 on p300 seems to be contrary to that of SIRT1. It deacetylates lysine residues in the catalytic domain of p300 leading to inhibition of transcription. Nicotinamide, a competitive inhibitor by product of the SIRT deacetylation reaction, was sufficient to prevent deacetylation of acetylated p300 [93]. In turn, p300 inhibits SIRT2 through acetylation, attenuating its negative influence on p53 protein [94]. The influence of different SIRTs on p300 and NF-κB activity is not fully determined. The measurement of selected SIRTs substrates in vivo such as, e.g., p53 or NF-κB, and other, remains an important tool for evaluating changes in SIRTs activity and give more light and understanding to complex processes in which SIRTs are involved.

## 2. Sirtuins in Selected Pathological States

**Figure 4:**
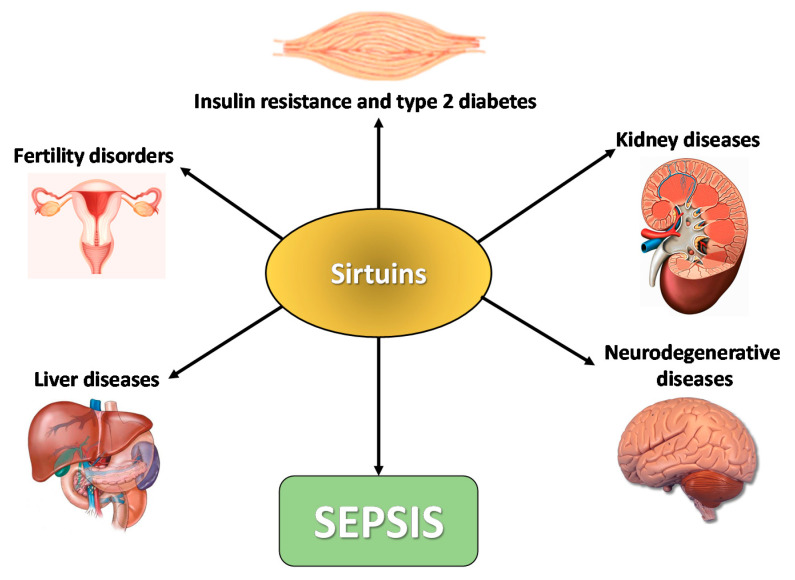
The enzymatic effect of SIRTs on selected organs. Modification according to Giblin et al. [103].

The wide spectrum of interactions and the multitude of cellular processes in which SIRTs take part, combined with the fact that disturbances in their expression and/or activity are observed in many pathological conditions occurring in the human body, point to the conclusion that SIRTs play a beneficial role in physiological states. Research on the influence of SIRTs as well as the level of their expression in homeostasis disturbances was made through clinical testing on animal models and in vitro cell cultures. A close relationship has been demonstrated between the degree of expression of SIRTs and diseases affecting many various internal organs [95,96,97,98,99,100,101,102]. The enzymatic activity of SIRTs on selected organs is schematically shown in Figure 4.

It has been proven that the degree of SIRTs expression varies depending on the type of abnormality in the body and the organ affected, as detailed in Table 2.

**Figure 5:**
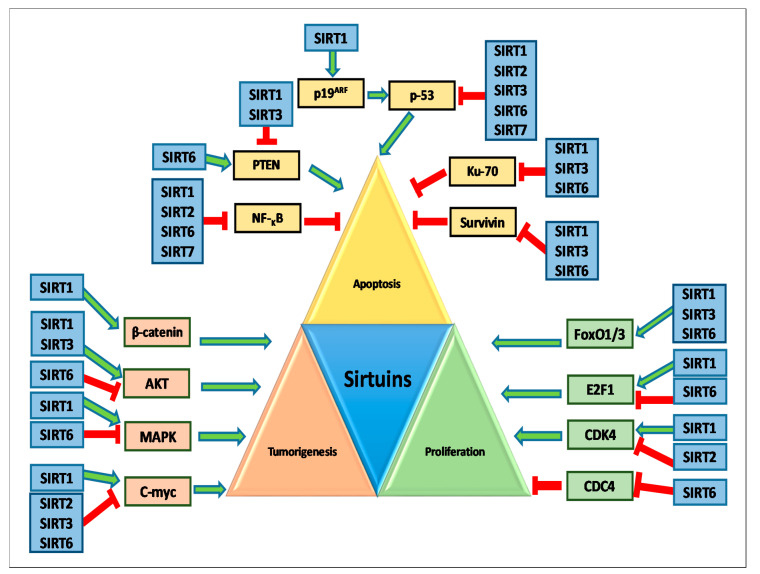
Role of SIRTs in cell proliferation, apoptosis, and tumorigenesis. AKT–protein kinase B; CDC4–cell division control protein 4; CDK4–cyclin-dependent kinase 4; C-myc–transcription factor; E2F1–transcription factor E2F1; FoxO1/3–forkhead family of transcription factors; Ku70–protein Ku; MAPK mitogen-activated protein kinase; NF-κB–transcription nuclear factor κB; PTEN–phosphatase and tensin homolog deleted on chromosome Ten; p19ARF–ARF tumor suppressor; p-53–protein 53; Survivin–baculoviral inhibitor of apoptosis; SIRT1-7–sirtuins 1-7; β-catenin–dual function protein; 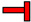
—inhibitory effect, 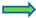
—activating effect. Modification based on Zhao et al. [169].

Increased expression of SIRTs can both promote the development of the disease and inhibit the progress of the pathological process in the body. In some disease states, an appropriate level of SIRTs expression may support treatment as well as be a prognostic factor in the ongoing disease process [109,112,114,118,132,138,148,165]. On the other hand, there is ample evidence that the levels of expression of a given sirtuin may be increased or decreased in the same pathology, which indicates a possible dual role for these proteins. For example, SIRT2 deficiency or inhibition with AGK2 appears to be protective mainly against acute liver injury and, conversely, SIRT2 overexpression aggravated liver damage, what suggest that SIRT2 exerts both protective and aggravating roles in cardiometabolic disorders, and its activation or inhibition depends on type and context of disorders [25]. Sirtuins play an important role in the post-translational modification of proteins. They cause changes in the chromatin structure, influencing the degree of acetylation/deacetylation of histones by direct participation in acetylation and deacetylation of lysine molecules present in histones. They can also change the activity of transcription factors and regulatory proteins, which additionally modulates changes in chromatin and affects the course of many processes in the cell, and thus the functioning of the organism. Sirtuins take part not only in post-translational modifications of proteins, but also in compensation mechanisms triggered in response to adverse environmental conditions and participate in epigenetic modifications that cause the development of many diseases, including type 2 diabetes, obstructive pulmonary disease, neoplastic, neurodegenerative or cardiovascular diseases as well [15,22,25]. Due to the fact that epigenetics is a rapidly developing branch of modern molecular biology, the epigenetic aspect of sirtuins action seems to be interesting, especially in the context of the role that sirtuins play in the pathogenesis of many diseases. Epigenetic changes include acquired and heritable chromatin modifications that regulate the expression and function of genes without affecting the DNA sequence, processes that perfectly match sirtuins. Sirtuins, apart from regulating the degree of histone acetylation by acting on various target substrates, are also involved in the regulation of the cell cycle, energy metabolism, the process of cell differentiation, apoptosis and cellular response to stress (e.g., oxidative, energetic or induced by ultraviolet radiation) [166,167,168]. Figure 5 shows how SIRTs activity affects cell proliferation, apoptosis and tumorigenesis.

## 3. Modulating Sirtuins Activity

Modulating the activity of sirtuins due to the multidirectional action of these deacetylases has also been the subject of numerous studies. Both activators and inhibitors of these enzymes can be divided into substances of natural origin (e.g., activator—resveratrol) and synthetic substances (e.g., inhibitor—MC3482). Sirtuin activators or inhibitors, i.e., compounds that either increase/decrease the rate of the enzymatic reaction or increase/decrease the affinity of the enzyme for the substrate, respectively, are becoming the object of increasing interest of many research teams due to their various functions described in Table 3 [25,170,171]. Since no activators or inhibitors are known for SIRT7 so far, it is not included in the table.

### 3.1. The Most Important SIRT Activators

#### 3.1.1. Cyanidin

Cyanidin is the major component of anthocyanins, commonly found in the Mediterranean diet. It is most abundant in red berries including bilberry, raspberry and cranberry. It has been documented that cyanidin exhibits anti-inflammatory effects in a variety of diseases [184]. Some authors [185,186] have suggested that anthocyanidins, including cyanidin, may play important roles in helping to reduce the risk of many age-related diseases. Cell culture and in vivo studies of anthocyanidins and their glycosylated counterparts (anthocyanins) revealed anticarcinogenic properties against colon, skin and lung cancer [185,186]. Studies by Cho at al. [187] on mice showed that mulberry cyanidin-3-glucoside inhibit the tumor proliferation and growth in the in vitro and in vivo model, indicating the inhibition of tumor progression [187]. Studies in a variety of cancer cells revealed that anthocyanins activate detoxifying enzymes, prevent cancer cell proliferation, induce cancer cell apoptosis, and have anti-inflammatory and antiangiogenic effects [185,186]. Cyanidin affected the expression of SIRT6-associated genes such as FoxO3α, Twist1 and GLUT1. FoxO3α belongs to the family of forkhead box transcription factors that plays an important role in regulating the expression of genes involved in cell growth, proliferation, differentiation and longevity. The deregulation of FoxO3 is involved in tumorigenesis. Previous studies reported that the FoxO3α gene is regulated by SIRT6, which forms a complex with FoxO3α in the nucleus and further induces the expression of genes involved in antioxidation [188].

#### 3.1.2. Curcumin

Curcumin is a phenolic compound extracted from the natural herb turmeric. It is obtained from turmeric rhizomes (*Curcuma longa* L.) and exists in two isoforms: ketone and enol. It is poorly absorbed in the small intestine, from where it is transported to the liver and then degraded [189]. Studies by Hu et al. [190] conducted on cell cultures showed that an increase in SIRT1 expression was observed in neoplastic cells (squamous cell carcinoma of the head and neck, FaDu and Cal27 cells) that were treated with curcumin. This activating effect was cancelled after preincubation with nicotinamide—the inhibitor of this deacetylase. Moreover, a decrease in the degree of acetylation of the lysine residues of the p53 protein, which is a substrate for SIRT1, was demonstrated, confirming the positive effect of curcumin on the activity of this sirtuin. It has also been shown that SIRT1 inhibit the expression of the NF-κB factor and proliferation of neoplastic cells, as evidenced by the reduced expression of the Ki-67 marker. The research of Sahin et al. [191] confirms that an increase in the expression of SIRT1 and the coactivator-1 α for the peroxisome proliferator-activated γ receptor (PGC1α) was observed in skeletal muscle cells of rats that were fed curcumin. In studies carried out on mice with myocardial damage, it was reported that curcumin reduces the intensity of myocardial fibrosis by activating SIRT1 through a mechanism dependent on this deacetylase [192]. This activator also influences lipid metabolism. The action of curcumin increases the concentration of the mRNA of ATP-binding cassette transporter protein (ABCA1), which is responsible for the transportation of excess cholesterol and phospholipids to macrophage-derived foam cells. A similar relationship was observed for the liver X receptor α (LXRα), suggesting that this receptor protein controls ABCA1 expression. It has been proven that the expression of AMP-activated protein kinase (AMPK) and SIRT1 increases in foam cells treated with curcumin [193]. Lin et al. [193] also showed that curcumin increases cholesterol efflux by increasing ABCA1 expression as a result of activating AMPK-SIRT1-LXRα signaling in foam cells derived from THP-1 macrophages. Moreover, after preincubation with an AMPK inhibitor, a decrease in the expression of LXRα, ABCA1 and SIRT1 was observed in these cells. This indicates that ABCA1 expression is dependent on the interaction of these compounds, which in turn is controlled by the action of curcumin [193]. Curcumin can also activate the PGC1α/SIRT3 signaling pathway to protect against mitochondrial impairment, and it can stimulate SIRT1 to have cardioprotective effects [192].

#### 3.1.3. Honokiol

Honokiol (HKL), a small molecular weight polyphenol, is an activator of SIRT3. HKL is derived from the magnolia tree. HKL’s anti-cancer activity has been studied extensively and it was found to be effective in several animal models of cancer, including lung, prostate, breast, colon and pancreatic cancers [194,195]. Besides its cytotoxicity in cancer, HKL was reported to induce a variety of cytoprotective activities, including anti-inflammatory, anti-thrombotic, anti-arrhythmic, neuroprotective, anti-oxidative, and anti-hypertrophic effects. Thus, HKL has the potential to act both as an antitumor and cytoprotective molecule [196,197].

#### 3.1.4. Luteolin

Luteolin (3′,4′,5,7-tetrahydroxylflavone), a natural flavonoid found in fruits, vegetables, and medicinal herbs, also exerts anti-inflammatory, anti-allergic, anti-cancer, anti-oxidant and other beneficial health effects [198]. Luteolin is also known to improve blood glucose, HbA1c, insulin levels and fatty acid metabolism-related gene expression [199]. Kim et al. [173] observed that SIRT1, SIRT3, SIRT6 and FoxO3a expression was decreased in hyperglycemia-induced monocytes. Conversely, the downregulated expressions of SIRT1, SIRT3, SIRT6 and FoxO3a were restored to levels similar to normoglycemic conditions by luteolin treatments. Luteolin treatments modulate SIRT1, SIRT3 and SIRT6 expressions under hyperglycemics conditions [173].

#### 3.1.5. Resveratrol

Resveratrol (RSV, 3,5,4’-trihydroxy-trans-stilbene) is a naturally occurring flavonoid that, due to its chemical structure, belongs to stilbens. It occurs in two optical isoforms: cis and trans, only the latter of which occurs naturally and is biologically active. Due to its protective effect, it is a natural phytoalexin, and is synthesized by plants in response to infections, structural damage, oxidative stress and excessive UV radiation. It occurs naturally in many plants and its highest concentration was found in the root of knotweed (*Reynoutria japonica*) as well as in various types of red wines, due to its presence in the skins of red grapes. It’s also present in lower concentrations in peanuts, berries, cranberries, strawberries, white hellebore, scots pine, rhubarb, orchids, tomatoes and cocoa. In humans, it is absorbed in the small intestine and metabolized in the liver, with both reactions catalyzed by cytochrome P450 enzymes [200,201].

RSV has been shown to be the primary natural activator of SIRT1. Using the Fluor de Lys kit (BioMol, Hamburg, Germany), Borra et al. [202] demonstrated the ability of RSV to cause an 8-fold increase in the activity of SIRT1, which was not detected for SIRT2. Moreover, it was observed that although SIRT1 stimulation was independent of acetylpeptide sequence, RSV activation was also related to the presence of a covalently attached fluorophore. Other fluorophore-containing media was shown to bind more closely to SIRT1 in the presence of RSV. RSV interacts with SIRT1 in allosteric activation. This compound activates SIRT1 through tree subunits—Res1, Res2 and Res3. These subunits act on SIRT1 to increase its ability to bind to the p53 protein. The activating properties of RSV are related to its interaction with the N-terminal part of the SIRT1 chain, and SIRT1 molecules lacking this fragment are not activated. The Res1 and Res2 subunits interact with both the N-terminal portion of the SIRT1 domain and its methylcoumarin substrate (p53-AMC) by forming hydrogen bonds. Res3, on the other hand, binds to the catalytic domain of deacetylase. Subsequently, the p53-AMC substrate is bounded by deacetylase at the site between the N- and C-terminus of SIRT1, and resveratrol enhances this interaction. Moreover, the presence of the glutamic acid residue located at position 230 (Glu230) has been shown to be important for the activation capacity of RSV. Lower activity of RSV is connected with conversion from glutamic acid to a lysine or alanine residue. Glu230 also interacts with an arginine residue located at position 446, present in the SIRT5 catalytic domain, resulting in an increase in the ability to activate this deacetylase [203,204]. Mancuso et al. [205] on SOD1G93A model of amyotrophic lateral sclerosis (ALS) mice revealed promising neuroprotective effects of RSV since it induces expression and activation of several neuroprotective pathways involving SIRT1 and AMPK in the ventral spinal cord. Their results demonstrate that microglial, but not astroglial reactivity, was significantly diminished with resveratrol administration compared to untreated SOD1G93A mice [205].

Studies by Pan et al. [179] demonstrated that resveratrol activates SIRT2 to deacetylate Prx1-27AcK, which significantly enhances its H_2_O_2_-reducing activity in HepG2 cells when using purified enzymes and/or substrates. The key role of H_2_O_2_ in carcinogenesis is supported by the tendency of cancer cells to have an elevated level of H_2_O_2_, which may play a key role in malignant transformation and explains many hallmarks of cancer, such as DNA damage and genetic instability. Conversely, an increase in H_2_O_2_-detoxifying enzyme activity can reduce the rate of cell proliferation and inhibit cancer metastasis [206]. Pan et al. [179] showed that small molecules targeting SIRT2 and Prx1 may modulate intracellular redox status in the therapeutic strategies for disorders related to aging. Whereas the studies of Eren et al. [207] revealed that resveratrol treatment induces premature senescence in human dermal fibroblasts, which is mediated by DNA damage and by the activation of p53-p21and Rb-p16 pathways. More importantly, concomitant decline in levels of SIRT1 and SIRT2 upon resveratrol treatment may be a cause for the induction of senescence, which is most likely mediated by a regulatory mechanism activated by DNA damage response [207]. Resveratrol is a polyphenol that has neuroprotective effects against many neurological disorders. This is confirmed by a study by Yan et al. [208], which investigated the potential protective effects of resveratrol in an in vitro ER stress model mimicked by tunicamycin treatment in neuronal HT22 cells. Authors reported that resveratrol exerts protective effects against tunicamycin-induced ER stress by regulating SIRT3-mediated autophagy in neuronal HT22 cells. These findings may reveal a new feature for the mechanism of resveratrol in neuroprotection, and provide further information regarding the role of SIRT3 in autophagy regulation [208]. SIRT5 can be activated by resveratrol and piceatannol (a resveratrol metabolite that carries an additional hydroxyl group), with a potency comparable to that reported for SIRT1 [209].

#### 3.1.6. SRT1720

SRT1720 is a synthetically produced chemical compound that belongs to polyphenols and is a derivative of RSV. It interacts with the catalytic domain of the enzyme, which reduces its activation energy for acetylated substrates and acts as an activator of this deacetylase. Although it significantly differs from RSV in molecular structure, it binds SIRT1 at the same target site and interacts with it through allosteric activation. It has been shown that SRT1720 binds to the SIRT1 substrate-peptide complex at the amino terminus of the catalytic domain and lowers the Michaelis constant for acetylated substrates. In addition, it has a much greater efficiency of SIRT1 activation (about 1000 times stronger than RSV) [175,210]. The ability to increase the catalytic activity of SIRT1 by SRT1720 was demonstrated by Chauhan et al. [211]. Studies conducted on multiple myeloma cells indicated an increase in apoptosis induced by the activation of caspases 8 and 9 as well as inhibition of the expression of another SIRT1 substrate—the NF-κB factor. The action of SRT1720 on myeloma cells also increases the concentration of ATM kinase (ataxia-telangiectasia mutated kinase) and increases its ability to interact with the effector kinase involved in DNA repair—checkpoint kinase 2 (CHEK2)—which leads to cell death induced by this complex. This activator inhibits the growth of multiple myeloma cells, as confirmed through in vivo studies by lowering the expression of the Ki-67 marker—an indicator of the cell’s ability to proliferate. It has been reported that, in Zucker rats (fa/fa type), by affecting SIRT1, this activator decreased the fasting glucose concentration and improved the insulin sensitivity of cells [210]. Administration of SRT1720 reduces the degree of acetylation of one of the substrates for sirtuin 1—PGC1α without affecting the concentration of this deacetylase in cells. As a consequence, decreased lipid storage in mouse liver cells, decreased aspartate aminotransferase activity, and the expression of genes of other enzymes involved in lipid metabolism, such as sterol regulatory element-binding protein 1 (SREBP-1c) and PGC1α, was observed. In the hepatocytes of mice treated with SRT1720, fat synthesis was decreased and lower concentration of free fatty acids, triglycerides and cholesterol was also noted [212].

#### 3.1.7. Quercetin

Quercetin (5,7,3′,4′-flavon-3-ol), is an organic polycyclic compound that belongs to flavonoids, the most abundant group in the world of plants. It belongs to the class of flavonols (3,4-diols flavan). It occurs, among others, in tea, fruit (apples, dark grapes, black currant, chokeberry), vegetables (e.g., onion, broccoli), herbs such as horsetail and St. John’s wort, and the flowers of e.g., hawthorn [213]. Due to its chemical structure, it belongs to the group of glycosides (e.g., rutin and rutoside), but it can also be present in free form as an aglycone. It is formed in a multistage reaction pathway from l-phenylalanine [214]. In the form of an aglycone, it is absorbed in the entire intestine, while glycosides can only be absorbed in the large intestine, where they undergo changes under the influence of enzymes produced by present bacteria [213]. It has been shown that short-term administration (7 days) of quercetin (12.5 mg/kg or 25 mg/kg) to mice results in an increase of the concentration of PGC1α mRNA and SIRT1, the expression of mtDNA, and cytochrome C concentration in skeletal muscle cells and in mice brains [172]. Moreover, it has been proved that administration of this compound to mice fed a high-fat diet for 12 weeks influenced not only the parameters of lipid and carbohydrate metabolism, but also inhibited the infiltration of macrophages into adipose tissue and suppressed the inflammatory process by acting on the AMPKα1/SIRT1 pathway [215]. In rats with streptozotocin-induced diabetes, after 12 weeks of administration of low or high doses of quercetin (10 mg/kg and 50 mg/kg, respectively), there was an improvement in lipid and carbohydrate metabolism parameters, the expression and hepatic activity of SIRT1 were increased, and the serine/threonine (Akt) protein kinase signaling pathway was activated by phosphorylation and deacetylation. This indicates that the beneficial effect of quercetin on the disturbance of glucose and lipid metabolism is probably related to the increased activity and concentration of the SIRT1 protein and its influence on the Akt signaling pathway [216]. Studies by Kamelo et al. [217] have shown that in liver cells of rats subjected to cytotoxic action of d-galactosamine/lipopolysaccharide complex (d-GalN/LPS), administration of this activator increased the expression of SIRT1 to a greater degree than observed after administration of its synthetic analogue—SRT1720, as evidenced by lower levels of liver damage markers (aminotransferases and bilirubin).

Quercetin has been shown to modulate SIRT6 in addition to SIRT1. Two quercetin derivatives, diquercetin and 2-chloro-1,4-naphthoquinone-quercetin, have been identified as promising SIRT6 inhibitors. 2-Chloro-1,4-naphtoquinone-quercetin also showed potent inhibition against SIRT2. Diquercetin increased the Km value of NAD^+^, whereas 2-chloro-1,4-naphthoquinone-quercetin increased the value of the acetylated substrate. Molecular docking studies suggest that diquercetin prefers the binding site of the nicotinamide (NAM) moiety, whereas 2-chloro-1,4-naphtoquinone-quercetin prefers to dock into the substrate binding site. The results of in vitro studies and molecular modelling indicated that diquercetin competes with nicotinamide adenine dinucleotide (NAD^+^), whereas 2-chloro-1,4-naphthoquinone-quercetin competes with the acetylated substrate in the catalytic site of SIRT6 [182].

### 3.2. The Most Important Sirtuin Inhibitors

#### 3.2.1. Cambinol

Cambinol inhibits NAD^+^-dependent deacetylase activity of human SIRT1 and SIRT2. It has weak inhibition against SIRT5 and no inhibition against SIRT3. Consistent with the role of SIRT1 in promoting cell survival during stress, inhibition of SIRT1 activity with cambinol during genotoxic stress leads to hyperacetylation of key stress response proteins and promotes cell cycle arrest. Cambinol exerts antitumor activity in vitro and in mouse xenograft studies [184]. Cambinol markedly decreases aromatase levels in human breast cancer cells by inhibiting SIRT1-mediated deacetylation and transcription activity of estrogen-related receptor α [218,219].

#### 3.2.2. Catechin

Catechin is a flavan-3-ol, a type of natural phenol and antioxidant. It belongs to the group of plant flavanols, which is part of the chemical family of flavonoids. Flavonoids are polyphenolic secondary metabolites synthesized by plants and fungus with various pharmacological effects [220]. Catechins hit multiple targets in cancer therapy and are also found to be useful in man-aging multidrug-resistant tumors. They have been shown to suppress several key pathways linked to oncogenesis, including those involved in cell survival, proliferation, and invasion, along with angiogenesis. They are also helpful against disorders involving lipid and glucose metabolism, such as type 2 diabetes and obesity, and could alleviate the risk of cardiovascular diseases [220]. Catechins have been shown to modulate the activity of a NAD^+^-dependent histone deacetylase, SIRT6. In the study by Rahnasto-Rilla et al. [184], catechins exhibited inhibition activity against SIRT6-catalyzed H3K9Ac deacetylation.

#### 3.2.3. MC3482

MC3482 is a synthetic sirtuin inhibitor that is effective in inhibiting SIRT5. After the application of this inhibitor, Polletta et al. [183] observed an increase in ammonia concentration in cells with a similar intensity as in cells with silenced expression of the SIRT5 gene. SIRT5 participates in the transformation of ammonia in the urea cycle by regulating carbamoyl phosphate synthetase I in liver and kidney cells [183].

#### 3.2.4. Nicotinamide

Nicotinamide (nicotinic acid amide) is formed as a product of NAD^+^ transformations under the influence of enzymes (e.g. SIRTs), nicotinamide nucleoside phosphorylase, or nicotinamide phosphoribosyltransferase. The products of nicotinamide metabolism in the body are nicotinamide n-oxide or n-methylnicotinamide [221]. Nicotinamide has been found to non-competitively hinder SIRT1 activity, as no competition of this inhibitor with the NAD^+^ molecule for the sirtuin 1 binding site was observed. However, nicotinic acid did not show the ability to inhibit SIRT1 [176]. In prostate neoplastic cells, the increased expression of SIRT1 compared to unchanged cells was diminished by the action of nicotinamide. Moreover, inhibition of the proliferation and growth of neoplastic cells was noted. The same effect was detected in cells in which the expression of this deacetylase was inhibited by the action of shRNA [222]. Studies carried out on cells from patients with chronic lymphocytic leukemia showed an augmented concentration of SIRT1 mRNA, compared to the B lymphocytes of healthy people. As a consequence of nicotinamide influence on these cells, the activity of SIRT1 is repressed and the apoptosis process is hyperactivated. Such a relationship was not detected during the suppression of p53 protein, which is a substrate for SIRT1. It seems that nicotinamide positively influences the induction of p53-dependent apoptosis by constraining the activity of SIRT1 [223].

#### 3.2.5. Salermide

Salermide (n-{3-[(2-hydroxy-naphthalen-1-ylmethylene)-amino]-phenyl}-2-phenyl-propionamide) is a reverse amide with a potent in vitro inhibitory effect on SIRT1 and SIRT2. It induces massive apoptosis in cancer but not in non-transformed cultured cells. The apoptotic effect of salermide is in part due to the reactivation of proapoptotic genes that are epigenetically repressed by SIRT1 exclusively in cancer cells [224].

#### 3.2.6. Selisistat

Selisistat (6-chloro-2,3,4,9-tetrahydro-1H-carbazole-1-carboxamide) is a selective SIRT1 inhibitor. It is over 200 times more selective towards SIRT1 compared to SIRT2 and SIRT3. It has been shown to inhibit the deacetylation of several SIRT1 substrates both in vitro and in vivo. This compound shows cyto- and neuroprotective activity in Huntington’s disease in both in vitro and in vivo models [225].

#### 3.2.7. Sirtinol

Sirtinol is a synthetic chemical compound and specific sirtuin inhibitor. In vitro studies by Grozinger et al. [180] confirmed that the action of sirtinol resulted in a reduction of SIRT2 activity in yeast cells, while it had no effect on the activity of histone deacetylases. Cells treated with sirtinol for 24 and 48 h showed a reduced ability to proliferate. Moreover, the action of sirtinol leads to a drop in SIRT1 activity in relation to its substrate FoxO1 in the nucleus and the cytoplasm of these cells, which results in an increase in the degree of acetylation of this factor and the inhibition of its activity. The factor FoxO1 induces cell apoptosis, which leads to inhibition of the growth of neoplastic cells [222]. Exposing breast cancer cells to sirtinol causes a significant inhibition of their proliferation and stimulates them to initiate the process of cell death. This compound shows a greater inhibitory efficiency against SIRT1 than SIRT2. It interacts with SIRT1 by forming hydrogen bonds with Gln345 and His363 residues, while with SIRT2 it interacts by forming hydrogen bonds with Gln167. As an outcome of its inhibitory action, sirtinol contributes to an increase in the degree of acetylation of the lysine residue located at position 382 of the p53 protein, which results in its activation [177].

#### 3.2.8. SirReal2

SirReal2 belongs to the family of aminothiazoles and is a potent and selective inhibitor of SIRT2—a NAD^+^-dependent lysine deacetylase that has been implicated in the pathogenesis of cancer, inflammation and neurodegeneration. SirReal2 inhibits SIRT2 only and has very little effect on the activities of SIRT3-5. The activities of SIRT1 and SIRT6 are slightly affected at higher SirReal2 concentrations, making SirReal2 one of the most selective sirtuin inhibitors [181].

#### 3.2.9. Suramin

Suramin is an organic compound that is mainly used in the treatment of African sleep sickness caused by Trypanosoma and HIV infection [226]. It also shows antiproliferative and antiviral activity [7]. Studies by Schuetz et al. [227] showed that suramin has the ability to inhibit SIRT5 as a result of interaction with the substrate binding site for SIRT5 and with NAD^+^.

#### 3.2.10. Tenovin

Tenovin is a bioactive small molecule that can exist in two isoforms: tenovin-1 and tenovin-6. Tenovins have the ability to hinder the activity of SIRT1 and SIRT2 and to increase the degree of acetylation of the p53 protein [178]. Tenovin-6 has also been shown to increase the intensity of cell apoptosis in acute promyelocytic leukemia, thereby inhibiting cell growth. In addition, the use of this inhibitor in treatment increased the differentiation of cells from the granulocyte lineage, as well as the surge in the degree of α-tubulin acetylation—a substrate for SIRT2. This effect is related to the inhibition of SIRT2 activity, which is not observed in the case of SIRT1. On the other hand, in cells from the granulocyte lineage with improved SIRT2 expression, their differentiation is inhibited due to the action of tenovin-6 [228]. Studies by Nihal et al. [229] showed augmented expression of SIRT1 in Cutaneous T-cell Lymphoma. Inhibition of this deacetylase activity by shRNA resulted in an increase in the concentration of FoxO3 protein and in cell apoptosis. The same effect was obtained by the action of tenovin-1 on these cells. Additionally, this inhibitor increased the degree of p53 protein acetylation [229].

## 4. Conclusions and Future Perspectives

SIRTs are a family of multi-functional enzymes that can contribute to both strengthening and weakening the enzymatic activity of proteins and can also alter their expression. In our previous review we discussed the structure and biological functions of sirtuins, including the most important substrates for their catalytic activity and the role that sirtuins play in cellular processes [9]. The multidirectional catalytic activity of sirtuins makes them an interesting and important particles allowing for the understanding of many processes taking place in the cell. Understanding all the functions and mechanisms of sirtuins’ action, as well as the interactions between SIRTs and modulators of their activity, is a great challenge for researchers. The acquired knowledge gives the opportunity to better understand the changes taking place in the cell, both in physiology and in pathology. It is particularly important to understand the influence of sirtuins on individual elements of the regulation of the process of DNA transcription and repair, regulation of cellular metabolism pathways and respiratory processes in cells, which could constitute an important point of reference for future diagnostic and therapeutic application. Despite the best understood role of SIRT1 and SIRT6 in modulating cellular processes, a growing area of research is also the investigation other classes of these enzymes. The role of SIRT5 seems to be of particular interest, due to its ability to catalyze demalonylation, deglutarylation and desuccinylation reactions, as well as its differentiated intracellular localization (cytoplasm, cell nucleus, mitochondria) [9].

There are many compounds known to regulate the activity of SIRTs, including ones of natural origin, which gives them great potential for use in the treatment of various diseases. However, despite the large number of investigations, the results of which contribute to the broadening of knowledge in this field, the development of an appropriate therapy based on SIRTs is still a challenging prospect. SIRTs regulate so many cellular processes that changing their activity can positively affect one process while at the same time negatively regulating another. The interesting aspect of its action is its role in oxidative stress development and its regulation by the Nrf2 factor, which is still poorly understood. Hence, it is extremely important to continue research along this path as well as examine the therapeutic use of SIRT while taking into account the role of activators and inhibitors that specifically influence their activity.

## Figures and Tables

**Table 1:** Human SIRTs—structure and target location.

Sirtuins	Location [10]	Protein Structure *
SIRT1	cell nucleus, cytoplasm	~82 kDa [11,12] 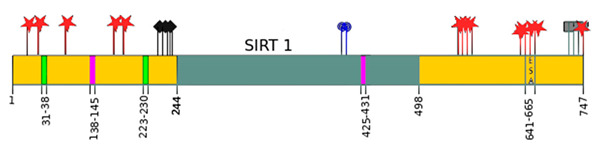
SIRT2	cell nucleus, cytoplasm	42 kDa [11] 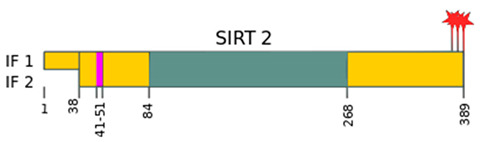
SIRT3	mitochondria	long form: 44 kDa [11] short form: 28 kDa [13] 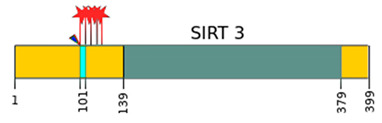
SIRT4	mitochondria	44 kDa [11] 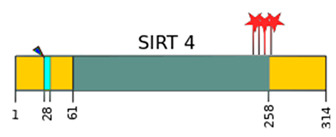
SIRT5	mitochondria, cell nucleus, cytoplasm	34 kDa [11,12] 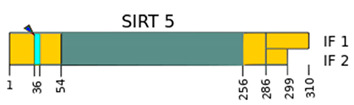
SIRT6	cell nucleus	39 kDa [11] 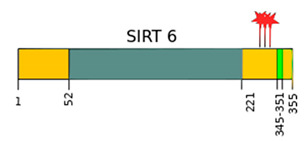
SIRT7	cell nucleus	48 kDa [11] 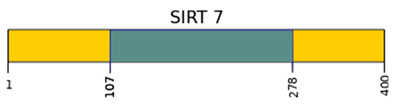

* Modification based on Flick and Lüscher [14] and Kratz et al. [9]. 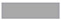
—catalytic domain NAD^+^- dependent, 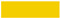
—small domain, 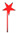
—phosphorylation site, 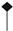
—methylation site, 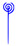
—nitrosylation site, 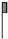
—sumoylation site, 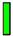
—Nuclear Localization Sequence (NLS), 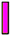
—Nuclear Export Sequence (NES), 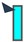
—mitochondrial cleavage site, IF1, IF2—two isoforms of SIRT2 and SIRT5.

**Table 2:** Participation of SIRTs in selected pathological disorders.

Sirtuins	Enzymatic Activity	Function [10]	Participation in Selected Pathological Conditions
SIRT1	NAD^+^-dependent deacetylase	formation of facultative and constitutive chromatin mitochondrial biogenesis fatty acid oxidation regulation of cholesterol and bile acid homeostasis	Increased expression: acute myeloid leukemia [104] chronic myeloid leukemia [105] cancer: stomach, colon [106,107] prostate [108] ovary, breast [109,110] melanoma [111] lungs [112] glioblastoma [113] Decreased expression: colon cancer [114] neurodegenerative diseases [17,25,97,115,116] cardiovascular diseases [25]
SIRT2	NAD^+^-dependent deacetylase	cell cycle regulation promoting lipolysis in adipocytes tumor suppression/promotion neurodegeneration	Increased expression: acute myeloid leukemia [117] prostate cancer [118] chronic myeloid leukemia [105] neurodegenerative diseases [17,25] Decreased expression: breast cancer [119] skin cancers [120] skin tumor [121] glioblastoma [122] cardiovascular diseases [25]
SIRT3	NAD^+^-dependent deacetylase	regulation of mitochondrial activity protection against oxidative stress tumor suppression	Increased expression: colon cancer [123] stomach cancer [124] Decreased expression: breast cancer [125] liver cancer [126,127] pancreatic cancer [128] chronic lymphocytic leukemia [129] female fertility disturbances [19] type 2 diabetes [16] neurodegenerative diseases [25,102] cardiovascular diseases [25]
SIRT4	ADP-ribosyltransferase	glucose metabolism catabolism of amino acids tumor suppression	Increased expression: esophageal cancer [130] breast cancer [131] thyroid cancer [132] myocardial hypertrophy [133] Decreased expression: stomach cancer [134,135] colon cancer [135,136] liver cancer [135] endometrial carcinoma [137] esophageal cancer [138]
SIRT5	NAD^+^-dependent deacetylase demalonylase desuccinylase	urea cycle fatty acid metabolism amino acid metabolism	Increased expression: non-small cell lung cancer [139] colorectal cancer [140,141] liver cancer [142] ovarian cancer [143] breast cancer [144] neurodegenerative diseases [25]
SIRT6	NAD^+^-dependent deacetylase ADP-rybozylotransferase	genomic stability/DNA repair glucose and lipid metabolism inflammation	Increased expression: multiple myeloma [145] acute myeloid leukemia [146] squamous cell carcinoma [120,147] liver cancer [148,149] prostate cancer [150,151] Decreased expression: colorectal cancer [152,153,154] breast cancer [155,156] liver cancer [98] bladder and prostate cancer [151] ovarian cancer [157] pancreatic cancer [158] neurodegenerative diseases [25] cardiovascular diseases [25] heart failure [159] cardiac fibrosis [160]
SIRT7	NAD^+^-dependent deacetylase	ribosome biogenesis tumor promotion	Increased expression: breast cancer [161] thyroid cancer [161,162] stomach cancer [151] colorectal cancer [151] prostate cancer [163] liver cancer [164] Decreased expression: pancreatic cancer [128,165]

**Table 3:** The most important known SIRT modulators.

Human Sirtuins	Activators	Inhibitors
SIRT1	curcumin [44] quercetin [172] luteolin [173] resveratrol [7,174] SRT1720 [175]	cambinol [7] nicotinamide [176] salermide [7] selisistat [7] sirtinol [177] tenovin-1 [178] tenovin-6 [178]
SIRT2	resveratrol [179]	cambinol [7] salermide [7] selisistat [170] sirtinol [180] SirReal2 [181] tenovin-1 [178] tenovin-6 [178] quercetin [182]
SIRT3	curcumin [170] honokiol [170] luteolin [173] resveratrol [170]	selisistat [170]
SIRT4	curcumin [170] resveratrol [170]	unknown
SIRT5	resveratrol [174]	cambinol [170] MC3482 [183] suramin [7]
SIRT6	cyanidin [184] quercetin [184] luteolin [184]	catechin [170] SirReal2 [170]
